# Exploratory predictive value of novel inflammatory biomarkers for microvascular obstruction after emergency revascularization in STEMI patients

**DOI:** 10.3389/fmed.2026.1946751

**Published:** 2026-10-13

**Authors:** Yang Dai, Longjin Chen, Zhe Zhang, Kaleem Ullah Shahzad, Qing Dai, Ying Tan, Xue Bao, Lina Kang, Biao Xu

**Affiliations:** 1 Department of Geriatrics, Nanjing Drum Tower Hospital, Affiliated Hospital of Nanjing University Medical School, Nanjing, China; 2 Department of Cardiology, Nanjing Drum Tower Hospital Clinical College of Nanjing Medical University, Nanjing, China; 3 Department of Cardiology, Yancheng City No.1 People’s Hospital, Yancheng First Hospital, Affiliated Hospital of Medical School, Nanjing University, Yancheng, China; 4 Department of Cardiology, Nanjing Drum Tower Hospital, Affiliated Hospital of Nanjing University Medical School, Nanjing, China; 5 Department of Cardiology, Cardiovascular Disease Center, Jiangsu Key Laboratory for Cardiovascular Information and Health Engineering Medicine, Nanjing Drum Tower Hospital, Medical School, Nanjing University, Nanjing, China

**Keywords:** blood tests, inflammatory markers, microvascular obstruction, predictive factors, ST-segment elevation myocardial infarction

## Abstract

**Purpose:**

To assess the associations of blood cell-derived inflammatory markers with the formation of microvascular obstruction (MVO) and their exploratory predictive performance in STEMI patients after PCI.

**Patients and methods:**

A retrospective analysis was conducted based on a prospective cohort, which included the patients with ST-segment elevation myocardial infarction (STEMI) who underwent emergency percutaneous coronary intervention (PCI) at Nanjing Drum Tower Hospital. All patients underwent cardiac magnetic resonance imaging within 7 days post-surgery, and were classified into MVO and non-MVO groups based on the examination results. Emergency and first post-PCI routine blood test and other clinical data were collected from patients, and various cell ratios were calculated, including the monocyte-to-lymphocyte ratio (MLR), neutrophil-to-lymphocyte ratio (NLR), platelet-to-lymphocyte ratio (PLR), systemic immune-inflammation index (SII), systemic inflammatory response index (SIRI), and aggregate index of systemic inflammation (AISI). The risk factors for MVO were evaluated by univariate and multivariate logistic regression analysis.

**Results:**

A total of 311 patients with STEMI were included. Among them, 198 developed MVO and the other 113 were MVO free. After adjustment for clinical covariates, higher post-PCI WBC, NEU, SIRI and NLR and greater peri-PCI eosinophil decline were associated with MVO. In the correlation-reduced joint analysis, post-PCI SIRI retained the most robust association.

**Conclusion:**

Postoperative WBC, NEU, SIRI, NLR, *Δ*EOS, *Δ*EOS% showed exploratory predictive performance for the formation of MVO after PCI in STEMI patients, and post-PCI SIRI showed the most robust association after accounting for biomarker intercorrelation.

## 1 Introduction

Acute myocardial infarction (AMI) is caused by insufficient blood supply to the heart, leading to ischemia and death of myocardial cells, and is a serious cardiac event resulting from coronary artery disease. ST-segment elevation myocardial infarction (STEMI) is the most acute form of myocardial infarction, which, in most cases, is caused by a complete occlusion of the coronary artery (1). Within a limited time window, direct percutaneous coronary intervention (PCI) or the early reperfusion with thrombolytic drugs is the most effective method to save ischemic myocardium and limit the area of infarction (2). However, evidence suggests that even with rapid revascularization, cardiac magnetic resonance (CMR) imaging shows myocardial microvascular obstruction (MVO) in more than half of the patients with STEMI (3–5), and it is closely associated with poor prognosis (6–9), however the mechanism of MVO formation is still not fully understood (10).

Substantial evidence indicates that immune-inflammatory responses play a significant role in the pathogenesis of myocardial infarction and are closely associated with clinical prognosis (11, 12). Peripheral blood inflammatory cell counts and their derived indexes can reflect the level of inflammation and have been widely used in clinical practice (12, 13). A complete blood cell count provides quantity, percentage, and morphological information for various cells, such as white blood cells (WBC), neutrophils (NEU), monocytes (MON), eosinophils (EOS), basophils (BAS), platelets (PLT), and lymphocytes (LYM), and is low-cost and easily available. Novel inflammatory markers derived from whole blood cells, including the monocyte-to-lymphocyte ratio (MLR), neutrophil-to-lymphocyte ratio (NLR), platelet-to-lymphocyte ratio (PLR), systemic immune-inflammatory index (SII), systemic inflammation response index (SIRI), and the aggregate index of systemic inflammation (AISI), can provide predictive value for the prognosis of coronary artery disease (14–18). Although NLR, PLR, and other hematologic inflammatory indices have been associated with angiographic no-reflow and adverse outcomes after STEMI/ACS (19, 20), and SIRI and AISI have shown prognostic value in patients undergoing PCI (14, 15), comparative evidence for multiple cell-derived indices, particularly SIRI and AISI, in relation to CMR-defined MVO remains limited (10, 21). Therefore, this study aimed to compare pre- and post-PCI routine blood cell-derived inflammatory indices in STEMI patients and to evaluate their associations and exploratory predictive performance for the formation of MVO.

## 2 Methods

### 2.1 General methods

This study is a retrospective analysis based on a prospective coronary artery disease database, involving patients diagnosed with STEMI and undergoing emergency PCI surgery at Nanjing Drum Tower Hospital during July 2018 and June 2023. Inclusion criteria: 1. Diagnosed with STEMI according to the fourth edition of the ‘Global Myocardial Infarction Definition’ (2018) (22); 2. Underwent PCI treatment within admission and had a CMR examination within one week (5d ± 2d) post-surgery; 3. PCI treatment strategies, methods, and hemodynamic assessments conform to the 2021 ACC/AHA/SCAI guidelines for coronary revascularization (23); 4. Had routine blood tests during emergency and within 24 h post-surgery. Exclusion criteria: 1. Patients with acute or chronic infections, severe autoimmune diseases, hematologic disorders, malignant tumors, or end-stage liver or kidney failure at admission; 2. History of previous myocardial infarction; 3. Contraindications to CMR or inability to cooperate. Ultimately, 311 patients were included in the analysis (Figure 1).

**Figure 1:**
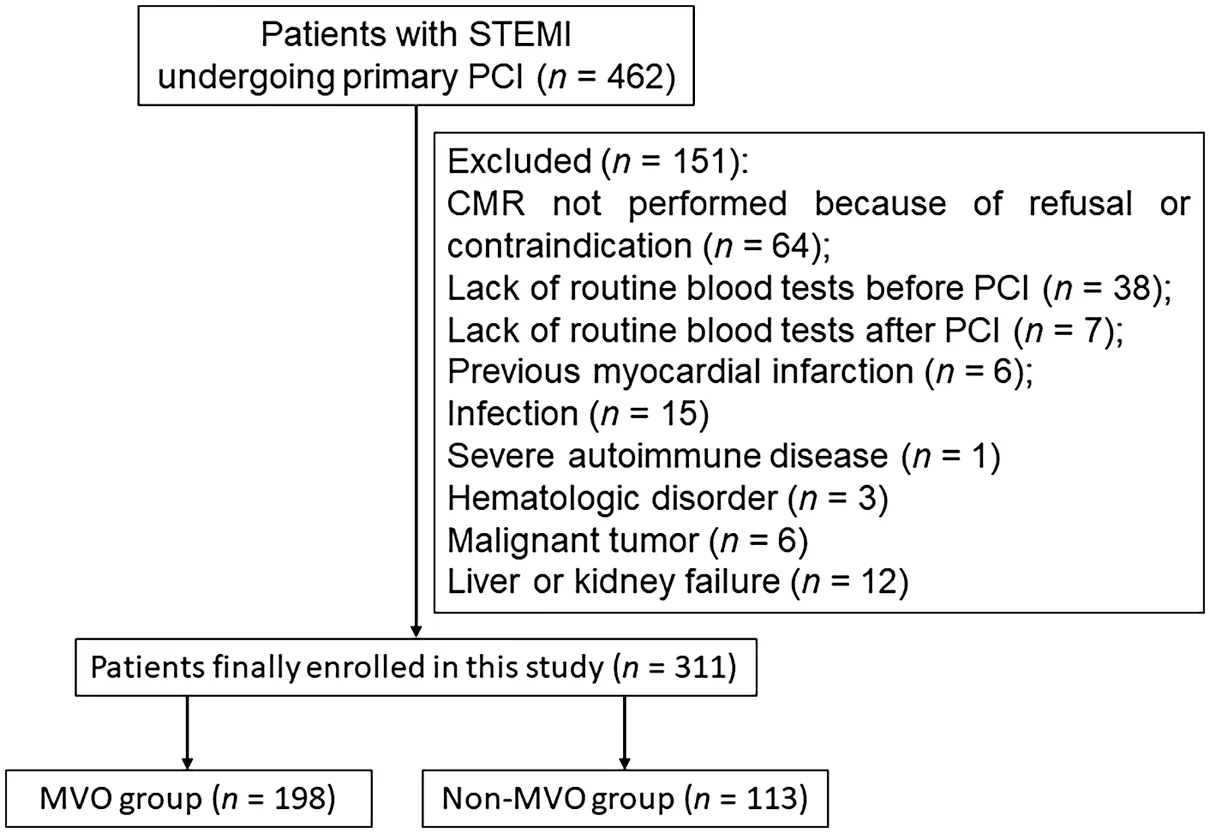
Flowchart onf patient screening and study inclusion.

Collection of patient data included: 1. Basic information: (1) General information: age, gender, height, weight, admission blood pressure, heart rate, cardiac function (Killip classification), etc.; (2) Current and past medical history: time of first onset and first medical contact, location of myocardial infarction, history of hypertension, diabetes, coronary artery disease, stroke, etc.; presence of angina symptoms; smoking and drinking history; family history of coronary artery disease, diabetes, hypertension; history of cardiovascular medication [antiplatelet drugs, anticoagulants, statins, *β*-blockers, angiotensin-converting enzyme inhibitors (ACEI)/angiotensin II receptor blockers (ARB), nitrates, calcium channel blockers (CCB), etc.]. 2. Laboratory examinations: Pre-PCI creatine kinase(CK), creatine kinase-MB (CKMB), troponin T (TNT), B-type natriuretic peptide (BNP), D-dimer; pre-PCI and within 24 h post-PCI routine blood tests; calculation of novel inflammatory markers, calculated as follows: MLR = monocyte count/lymphocyte count, NLR = neutrophil count/lymphocyte count, PLR = platelet count/lymphocyte count, SII = neutrophil count × platelet count/lymphocyte count, SIRI = neutrophil count × monocyte count/lymphocyte count, AISI = neutrophil count × monocyte count × platelet count/lymphocyte count, and changes were calculated as post-PCI minus pre-PCI values. 3. Surgical related data, echocardiogram and magnetic resonance data: (1) the culprit vessel shown during coronary angiography, presence of collateral circulation, number of stents implanted, post-stent dilation and thrombus aspiration, postoperative TIMI frame count. (2) Echocardiogram: left ventricular ejection fraction (LVEF), measurement of left atrial diameter (LAD), aortic diameter (AoD), left ventricular end-diastolic diameter (LVDd), valvular regurgitation, etc.; (3) Cardiac magnetic resonance: Using left ventricular short-axis delayed gadolinium enhancement (LGE) images to analyze the presence of microvascular obstruction (MVO), with the infarcted area showing high signal on myocardial tissue and MVO being described as a low signal area within the high signal region of the myocardium.

### 2.2 Statistical methods

Participants were grouped based on the occurrence of MVO. Continuous variables were described using mean ± standard deviation or median (interquartile range) depending on their distribution. For normally distributed quantitative data, the Student's t-test was used for comparisons between groups, while the Mann–Whitney U test was used for non-normally distributed data. Categorical variables were presented as numbers (percentage) and compared using the Chi-square test or Fisher's exact test. Univariate logistic regression analysis was used to identify factors associated with MVO. Multivariate logistic regression models were employed, with inflammatory factors as independent variables (one at a time) and the occurrence of MVO as the dependent variable. Other baseline variables that were significant in univariate analysis were adjusted for as covariates. The variance inflation factor was used to check for potential multicollinearity among independent variables. Pearson or Spearman correlation was used to analyze the associations between inflammatory indexes. In a sensitivity analysis, we performed correlation-based reduction (|r| ≥ 0.70) to retain representative markers from highly correlated biomarkers. False discovery rate (FDR) adjustment was then applied to these markers in separate models. All representative markers retained after correlation-based reduction were also entered into a single stepwise multivariable model. The variance inflation factor (VIF) was used to assess potential multicollinearity among independent variables. To evaluate the predictive performance of the inflammatory markers, we assessed discrimination using the area under the receiver operating characteristic curve (AUC) and calibration using the Hosmer-Lemeshow test. To quantify incremental predictive value, the multivariable model with and without the markers were compared, and change in AUC (*Δ*AUC), Integrated Discrimination Improvement (IDI), and Net Reclassification Improvement (NRI) were then calculated.

All data processing and analysis in this study were conducted using the statistical software SAS 9.4, with a two-sided *p*-value of <0.05 considered statistically significant. Other baseline variables that were significant (*p* < 0.05) in univariate analysis were adjusted for as covariates.

## 3 Results

### 3.1 Comparison of baseline data between the MVO group and the non-MVO group

A total of 311 patients were enrolled in this study, including 198 in the MVO group and 113 in the non-MVO group. As shown in Table 1, compared with the non-MVO group, the systolic blood pressure of the MVO group at admission was significantly lower than that of the non-MVO group (*p* = 0.047), and the heart rate at admission was significantly higher than that of the non-MVO group (*p* = 0.047). There was no statistically significant difference in other baseline data (*p* > 0.05).

**Table 1:** Baseline data of patients.

Parameter	*N*	Non-MVO group	MVO group	*p* value
age (years)	311	60.86 ± 12.63	58.38 ± 12.69	0.099
gender (male)	311	97 (85.84)	177 (89.39)	0.352
BMI (kg/m^2^)	267	24.49 (22.78–26.67)	25.25 (22.84–27.34)	0.303
smoking history	309	69 (61.61)	127 (64.47)	0.616
drinking history	307	27 (24.11)	55 (28.21)	0.435
diabetes	311	72 (63.72)	133 (67.17)	0.536
hypertension	311	59 (52.21)	113 (57.07)	0.407
history of coronary heart disease	308	1 (0.90)	3 (1.52)	1.000
family history of coronary heart disease	308	17 (15.32)	31 (15.74)	0.922
hyperlipidemia	311	60 (53.10)	126 (63.64)	0.068
stroke	311	20 (17.70)	20 (10.10)	0.054
angina pectoris	300	54 (50.00)	87 (45.31)	0.435
antiplatelet drugs	291	5 (4.76)	11 (5.91)	0.679
anticoagulants	290	0 (0.00)	2 (1.08)	0.537
statins	290	4 (3.81)	14 (7.57)	0.202
β receptor blockers	291	3 (2.86)	14 (7.53)	0.103
ACEI/ARB drugs	291	17 (16.19)	38 (20.43)	0.375
nitrates	291	4 (3.81)	2 (1.08)	0.193
CCB	295	16 (15.09)	40 (21.16)	0.202
heart rate at admission (beats/min)	311	80.89 ± 14.84	84.47 ± 15.43	0.047
systolic blood pressure at admission (mmHg)	311	128.40 ± 19.93	123.85 ± 18.92	0.047
diastolic blood pressure at admission (mmHg)	311	78.11 ± 13.93	76.68 ± 13.21	0.371
cardiac function (Killip)	311	1 (1–1)	1 (1-1)	0.798
anterior wall myocardial infarction	311	53 (46.90)	98 (49.49)	0.66

### 3.2 Auxiliary examination and emergency PCI related indexes in MVO group and non-MVO group

As shown in Table 2, compared with the non-MVO group, the MVO group had larger left ventricular end-diastolic diameter (LVDd) (*p* < 0.001), larger left atrial diameter (LAD) (*p* = 0.011) and lower Left ventricular ejection fraction (EF%) (*p* < 0.001). Compared with the non-MVO group, more patients in the MVO group used nitroprusside during surgery (*p* = 0.043) and a greater proportion of patients received thrombus aspiration (*p* = 0.001).

**Table 2:** Auxiliary examination and emergency PCI related indexes.

Parameter	*N*	Non-MVO group	MVO group	*p* value
CK (U/L)	187	154.00 (93.00–405.00)	192.50 (96.50–456.50)	0.434
CKMB (U/L)	195	23.00 (13.00–47.00)	25.00 (13.00–60.00)	0.602
TNT (ng/mL)	198	0.17 (0.02–0.62)	0.10 (0.03–0.53)	0.759
BNP (pg/mL)	259	64.80 (20.00–179.00)	51.15 (15.80–174.00)	0.282
D dimer (ug/mL)	185	0.32 (0.21–0.50)	0.28 (0.19–0.44)	0.196
IVSTd (cm)	303	0.90 ± 0.14	0.90 ± 0.14	0.980
LVDd (cm)	311	5.20 (5.00–5.40)	5.32 (5.10–5.60)	＜0.001
LVPWT(cm)	298	0.87 ± 0.12	0.87 ± 0.11	0.863
AoD (cm)	299	3.23 ± 0.31	3.17 ± 0.26	0.106
LAD (cm)	311	3.86 ± 0.31	3.97 ± 0.38	0.011
EF%	311	48.01 ± 5.3	45.85 ± 5.27	＜0.001
Intraoperative medication: nitroprusside	311	7 (6.19)	27 (13.64)	0.043
Intraoperative medication: nitroglycerin	311	107 (94.69)	180 (90.91)	0.229
Intraoperative medication: IIb/IIIa antagonists	311	49 (43.36)	92 (46.46)	0.597
post-stent expansion pressure	295	14.36 ± 5.60	14.64 ± 4.87	0.650
time from FMC to reperfusion (h)	287	1.27 (0.89–2.19)	1.12 (0.87–1.67)	0.102
time from onset to reperfusion (h)	304	5.18 (3.04–12.03)	5.22 (3.16–10.73)	0.706
collateral circulation formation	311	13 (11.50)	17 (8.59)	0.402
Culpritvessel (anterior descending artery or not)	311	51 (45.13)	99 (50.00)	0.409
thrombus aspiration	311	26 (23.01)	82 (41.41)	0.001

CK, creatine kinase; CKMB, creatine kinase isoenzyme MB; TNT, troponin T; BNP, B-type natriuretic peptide; IVSTd, end-diastolic interventricular septal thickness; LVDd, left ventricular end-diastolic diameter; LVPWT, left Posterior ventricular wall thickness; AoD, aortic diameter; LAD, left atrial diameter; EF, left ventricular ejection fraction; FMC, first medical contact.

### 3.3 Routine blood test and derived inflammatory indicators in MVO group and non-MVO group

As shown in Supplementary Table S1, compared to the non-MVO group, the MVO group had significantly higher preoperative WBC (*p* = 0.033), preoperative SIRI (*p* = 0.033), postoperative WBC (*p* = 0.001), postoperative NEU% (*p* = 0.005), postoperative NEU (*p* < 0.001), postoperative SII (*p* = 0.007), postoperative SIRI (*p* < 0.001), postoperative MLR (*p* = 0.015), postoperative NLR (*p* < 0.001), and postoperative AISI (*p* = 0.016). Postoperative LYM% (*p* = 0.004) and postoperative EOS% (*p* = 0.018) were significantly lower; compared to the non-MVO group, the MVO group showed a more pronounced decrease in postoperative EOS (*p* = 0.006) and EOS% (*p* = 0.005) from before surgery.

### 3.4 Analysis of the correlation between routine blood test-related inflammatory indexes

Correlation analysis indicated that there were varying degrees of correlation between inflammatory indexes, with correlation coefficients ranging from −0.94487 to 0.95556 (Figure 2).

**Figure 2:**
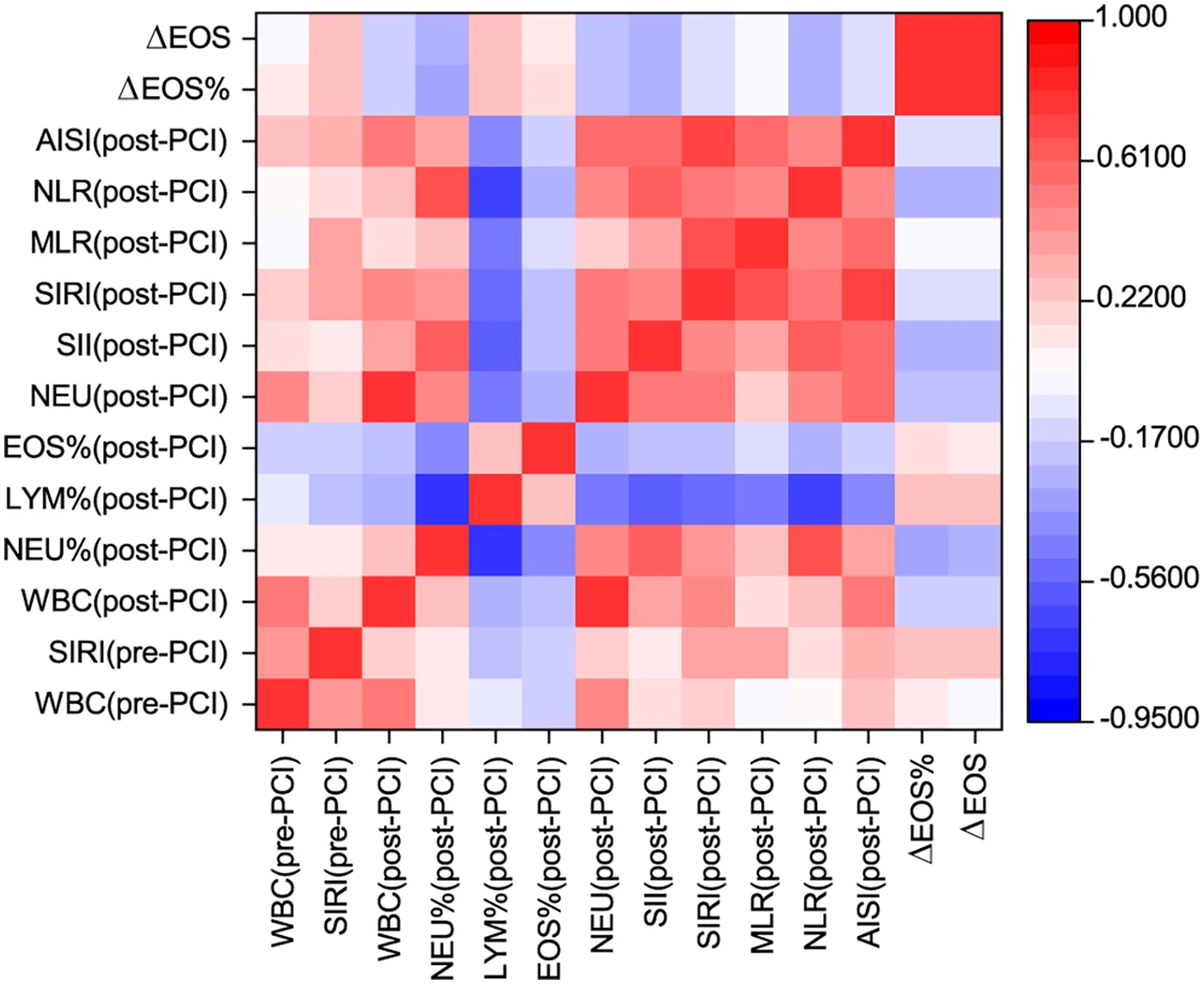
Heatmap of the correlation analysis of routine blood test-related inflammatory indexes.

### 3.5 Logistic regression analysis of the association between routine blood test-related inflammatory indexes and MVO

Univariate logistic regression showed that all routine blood test-related inflammatory indexes with baseline differences were associated with MVO. After further adjustment for other baseline factors with significant differences, such as LVDd, LAD, EF%, heart rate at admission, systolic blood pressure at admission, intraoperative use of nitroprusside, and thrombus aspiration, the results showed that higher postoperative WBC (*p* = 0.028), postoperative NEU (*p* = 0.016), higher postoperative SIRI (*p* = 0.024), higher postoperative NLR (*p* = 0.033), larger decrease in eosinophil count from preoperative to postoperative (*Δ*EOS, *p* = 0.029), and larger decrease in eosinophil percentage from preoperative to postoperative (*Δ*EOS%, *p* = 0.017) were independently associated with the formation of MVO (Table 3).

**Table 3:** Association analysis of routine blood test-related inflammatory indexes with MVO (per standard deviation change).

Parameter	Univariate regression analysis		Multivariate regression analysis	
	OR (95%CI)	*p* value	OR (95%CI)	*p* value
WBC (pre-PCI)	1.30 (1.02, 1.65)	0.034	1.16 (0.90, 1.50)	0.242
SIRI (pre-PCI)	1.29 (1.00, 1.66)	0.050	1.17 (0.90, 1.53)	0.237
WBC (post-PCI)	1.50 (1.17, 1.94)	0.002	1.35 (1.03, 1.76)	0.028
NEU% (post-PCI)	1.40 (1.10, 1.77)	0.006	1.25 (0.97, 1.61)	0.083
LYM% (post-PCI)	0.71 (0.56, 0.90)	0.005	0.80 (0.63, 1.03)	0.088
EOS% (post-PCI)	0.78 (0.61, 0.99)	0.038	0.83 (0.66, 1.06)	0.136
NEU (post-PCI)	1.60 (1.23, 2.07)	0.001	1.40 (1.06, 1.84)	0.016
SII (post-PCI)	1.43 (1.08, 1.88)	0.012	1.26 (0.94, 1.68)	0.117
SIRI (post-PCI)	1.67 (1.22, 2.29)	0.002	1.43 (1.05, 1.96)	0.024
MLR (post-PCI)	1.36 (1.04, 1.77)	0.024	1.22 (0.93, 1.62)	0.153
NLR (post-PCI)	1.62 (1.21, 2.18)	0.001	1.40 (1.03, 1.91)	0.033
AISI (post-PCI)	1.53 (1.13, 2.07)	0.006	1.35 (1.00, 1.84)	0.054
ΔEOS%	0.68 (0.51, 0.90)	0.007	0.69 (0.50, 0.94)	0.017
ΔEOS	0.67 (0.50, 0.90)	0.008	0.70 (0.51, 0.96)	0.029

WBC, white blood cells; SIRI, systemic inflammation response index; NEU, neutrophils; LYM, lymphocytes; EOS, eosinophils; SII, systemic immune-inflammatory index; MLR, monocyte-to-lymphocyte ratio; NLR, neutrophil-to-lymphocyte ratio; AISI, aggregate index of systemic inflammation.

### 3.6 Correlation-reduced and multivariable analyses of inflammatory markers associated with MVO

After correlation-based reduction, six markers (preoperative SIRI, postoperative WBC, postoperative eosinophil percentage, postoperative SIRI, postoperative NLR, *Δ*EOS%) remained significant in univariable analyses with FDR correction. When each marker was tested in separate multivariable model, four markers (postoperative WBC, postoperative SIRI, postoperative NLR, and *Δ*EOS%) retained their associations (*p* < 0.05), consistent with uncorrected results. In the stepwise multivariable model including all six retained markers, postoperative SIRI remained statistically significant [OR (95% CI), 1.50 (1.10, 2.05); *p* = 0.0103]. All VIF values across models ranged 1.04-2.90, indicating no substantial multicollinearity.

### 3.7 Exploratory predictive performance and incremental value of inflammatory biomarkers for MVO

The models with biomarkers alone demonstrated modest discrimination. SIRI showed a relatively good predictive performance with an AUC of 0.622. Calibration was satisfactory (Hosmer-Lemeshow test, *p* > 0.05). When added to the multivariable model, the inflammatory markers did not significantly improve overall discrimination nor yield a significant NRI. However, the IDI was statistically significant (Table 4).

**Table 4:** Performance of the biomarkers.

Parameter	WBC (post-PCI)	NEU (post-PCI)	SIRI (post-PCI)	NLR (post-PCI)	ΔEOS%	ΔEOS
biomarker alone						
AUC	0.600	0.618	0.622	0.612	0.574	0.566
calibration *p*	0.264	0.393	0.190	0.660	0.658	0.753
optimal cut-off values	10.700	6.900	4.350	5.636	−1.300	−0.080
sensitivity to specificity ratio	0.650	1.396	0.419	0.706	0.194	0.306
adding into the multivariable model						
ΔAUC	0.012	0.014	0.016	0.010	0.013	0.010
*p*	0.326	0.309	0.246	0.412	0.281	0.384
IDI	0.014	0.016	0.016	0.013	0.016	0.013
*p*	0.048	0.031	0.018	0.045	0.033	0.053
NRI	0.090	0.214	0.216	0.156	0.204	0.196
*p*	0.446	0.070	0.066	0.187	0.084	0.096

AUC, area under receiver operating characteristic curve; IDI, integrated discrimination improvement; NRI, net reclassification improvement.

## 4 Discussion

In this retrospective cohort, several post-PCI blood cell-derived inflammatory indices and peri-PCI eosinophil changes were associated with CMR-defined MVO after adjustment for clinical covariates. Because the inflammatory indices were strongly intercorrelated, the one-marker-at-a-time models should not be interpreted as demonstrating mutual independence among biomarkers. After correlation-based reduction and FDR correction, six representative markers remained significant in univariable analyses; when these markers were considered together, post-PCI SIRI remained statistically significant.

Although treatment methods have matured, acute myocardial infarction (AMI) remains a major cause of global morbidity and mortality, driving efforts to predict and improve prognosis (13–18, 24, 25). Inflammation drives atherosclerosis progression, influences MI repair/remodeling, and elevates cardiovascular risk in AMI patients (25–27). Chronic, mild inflammation may promote the progression of cardiovascular disease (28). Reducing inflammatory responses is thus explored as a therapeutic strategy, and whole blood cell counts are increasingly studied for their accessibility and predictive utility (13–18). Previous research has indicated that differential white blood cell (WBC) profiles yield stronger outcome predictions than total WBC, with the neutrophil-to-lymphocyte ratio (NLR) most strongly linked to mortality or infarction (29). Zhang's meta-analysis confirmed NLR predicts post-percutaneous coronary intervention (PCI) STEMI prognosis: higher ratios correlate with poorer outcomes (19). Park et al. specifically noted that post-PCI NLR is related to clinical outcomes, and NLR within 24 h after admission can better predict mortality compared to baseline or pre-PCI levels (30), which is consistent with our study results. The reason may be that the time after myocardial infarction is too short for the body to fully respond to the severity of STEMI, so the pre-PCI white blood cell spectrum may not fully reflect the severity of myocardial damage. Additionally, the postoperative white blood cell spectrum can also reflect myocardial damage during surgery (31), which is not reflected by the preoperative white blood cell spectrum. Furthermore, post-PCI CMR timing correlates with post-PCI NLR levels reflecting myocardial damage better than baseline values due to temporal alignment. Infiltrating neutrophils exacerbate plaque degradation and infarct enlargement via hydrolases, superoxide radicals, and cytotoxic metabolites. The aggregation of neutrophils and platelets can cause microvascular damage, thereby promoting the expansion of the infarct area and the formation of MVO (29). Additionally, studies have indicated that a reduction in circulating lymphocytes early after reperfusion is closely related to the development of MVO, as lymphocytes may homing to the microvasculature of the myocardium, producing inflammatory mediators, further increasing the infiltration of white blood cells and myocardial damage (32). NLR, which includes both neutrophil and lymphocyte types of white blood cells, is a single risk factor for predicting MVO, thus it has better predictive value. In patients with myocardial infarction undergoing PCI, a high PLR is associated with the occurrence of major adverse cardiovascular events (MACE) and is an independent risk factor for insufficient myocardial reperfusion (33). Even in patients with acute STEMI, PLR is related to the occurrence of no reflow after PCI (20). Studies have shown that an elevated MLR is an independent predictor of long-term MACE in patients with myocardial infarction (34). However, in this study, although univariate analysis suggested that an increased postoperative MLR is a risk factor for the formation of MVO, it was not found to be independently associated with the formation of MVO after adjusting for confounding factors. This may indicate that neutrophils are more closely related to the formation of MVO than monocytes and platelets.

SII and SIRI are considered more comprehensive and stable compared to NLR, PLR, and LMR. Research suggests that SII and SIRI are superior in predicting cardiovascular diseases compared to NLR, PLR, and LMR (35, 36). Notably, in addition to neutrophils and lymphocytes, SII includes platelets, while SIRI incorporates monocytes. Monocytes are primary cells in the formation of atherosclerotic plaques and directly participate in the development of atherosclerosis. They adhere to the endothelium, differentiate into macrophages, and then transform into foam cells by ingesting oxidized lipoproteins, which can activate various inflammatory signaling molecules and oxidative free radicals within the plaque (37, 38). Although activated platelets are involved in thrombus formation during atherosclerotic plaque rupture or endothelial erosion (39), considering that monocytes are the primary cells in plaque formation, they may have a closer relationship with the progression of atherosclerosis than platelets. Jin et al. found that elevated SII and SIRI increased the risk of stroke and all-cause mortality. However, the high risk of myocardial infarction was only independently associated with high SIRI, not with SII (16). Han et al. found that SIRI is an independent risk factor for major adverse cardiovascular events (MACE) (15). In this study, SIRI also demonstrated its good predictive value for MVO. AISI combines four indicators to reflect the overall state of the body and should have the capacity to reflect more information. Currently, there are few studies on AISI and the prognosis of myocardial infarction. Fan et al., through a follow-up of 1,558 patients undergoing PCI treatment for ACS, found that higher AISI and SIRI were associated with a higher risk of MACE (14). In this study, univariate analysis suggested that an increase in postoperative AISI was a risk factor for the formation of MVO. After adjusting for confounding factors in the multivariate analysis model, it was found that *p* was 0.054, with no significant statistical difference. This may also be related to our smaller sample size and selection bias. A recent study by Aygün et al. evaluated inflammatory and glycemic indices in STEMI and found that composite laboratory indices, particularly the neutrophil glycemic index, were associated with in-hospital mortality and showed useful ROC discrimination (40). Although that study evaluated a different endpoint from CMR-defined MVO, it further illustrates the potential value of routinely available composite laboratory markers for early STEMI risk stratification.

Furthermore, our study found that the more pronounced the postoperative decrease in EOS and EOS% compared to preoperative levels, the greater the likelihood of MVO formation. Marx et al. suggest that eosinophils contribute to the formation of atherosclerotic plaques by increasing the exposure of vascular von Willebrand factor on endothelial cells and enhancing platelet adhesion; during arterial thrombosis, eosinophils are rapidly recruited in an integrin-dependent manner and interact with platelets, inducing the formation of eosinophil extracellular traps (EETs) (41). EETs, modified by major basic proteins, can activate platelets, thus promoting thrombus formation. Rios-Navarro et al. found in a pig heart model that eosinophil counts rise after ischemia and drop rapidly after reperfusion; they also found that in STEMI patients, a rapid post-reperfusion drop in eosinophil counts correlates with more pronounced cardiac MRI-detected edema, microvascular obstruction, and infarct size, and a higher probability of cardiac events (death, reinfarction, or heart failure) during follow-up (21). This is consistent with our study results.

Several limitations should be acknowledged. First, this retrospective, single-center analysis of a prospective database is susceptible to selection bias, residual confounding, and limited generalizability, and it cannot establish causality. Although the patient-flow diagram transparently reports the screening and exclusion process, requiring both CMR within 7 days and complete paired pre-/post-PCI blood tests may have selected patients able to undergo more complete clinical evaluation. No *a priori* sample-size calculation was performed because this was a retrospective analysis of all eligible patients in the existing cohort; the modest sample size may limit the precision and stability of the estimated associations and performance measures. Second, although several measured clinical and procedural variables were considered, the models did not comprehensively adjust for all potential determinants of MVO, including initial TIMI flow, angiographic thrombus burden, and infarct size. The study period spanned 2018–2023; however, there were no major changes in guideline-directed primary PCI strategies or institutional practice during this period, and the principal procedural and adjunctive pharmacological approaches remained broadly consistent. Nevertheless, minor temporal variations in operator preference, devices, or supportive treatment cannot be completely excluded. Third, inflammatory markers were measured only before PCI and within 24 h after PCI. Because post-PCI blood samples were obtained after reperfusion, these biomarkers may partly reflect infarct severity and reperfusion-related inflammation rather than temporally antecedent predictors of MVO. The change-score models were not additionally adjusted for baseline EOS/EOS%, so the eosinophil-change findings should be considered exploratory. Fourth, multiple correlated biomarkers were evaluated. Although correlation-based reduction, FDR correction, and a joint multivariable sensitivity analysis were performed, residual type I error and model instability cannot be excluded. No independent internal or external validation was performed; consequently, the reported cut-offs and performance estimates may be sample-specific and require validation before clinical use.

## 5 Conclusion

Several post-PCI blood cell-derived inflammatory indices and peri-PCI eosinophil changes were associated with CMR-defined MVO after adjustment for clinical covariates. After accounting for biomarker intercorrelation, post-PCI SIRI showed the most robust association and the highest AUC among the evaluated markers, although overall predictive performance was modest and incremental predictive value was limited. These routine indices may have potential as inexpensive adjunctive risk-stratification or triage markers, but their predictive utility requires prospective multicenter validation.

## Data Availability

The original contributions presented in the study are included in the article/Supplementary Material, further inquiries can be directed to the corresponding authors.
