# Supplementary material for: Exploratory predictive value of novel inflammatory biomarkers for microvascular obstruction after emergency revascularization in STEMI patients
Source: Front Med (Lausanne). 2026 Oct 13;13:1946751. doi: 10.3389/fmed.2026.1946751 (PMC13710088; doi:10.3389/fmed.2026.1946751)
Supplement: Supplementary file 1 [file Table1.docx]

**Supporting Information**

**Exploratory predictive value of novel inflammatory biomarkers for microvascular obstruction after emergency revascularization in STEMI patients.**

Table S1. Routine blood test and derived inflammatory indicators

|  |  | Non-MVO group（N=113） | MVO group（N=198） | *p* value |
| --- | --- | --- | --- | --- |
| Pre-PCI | WBC(10^9^/L) | 10.31 ± 2.98 | 11.09 ± 3.14 | 0.033 |
|  | NEU% | 71.10 ± 12.75 | 72.14 ± 13.52 | 0.506 |
|  | LYM% | 21.41 ± 10.75 | 20.56 ± 11.98 | 0.531 |
|  | MON% | 6.24 ± 2.33 | 5.92 ± 2.18 | 0.229 |
|  | EOS% | 0.50 (0.20-1.10) | 0.50 (0.20-1.30) | 0.950 |
|  | BAS% | 0.31 ± 0.22 | 0.32 ± 0.20 | 0.864 |
|  | NEU(10^9^/L) | 7.48 ± 2.97 | 8.14 ± 3.14 | 0.068 |
|  | LYM(10^9^/L) | 2.09 ± 1.10 | 2.16 ± 1.28 | 0.642 |
|  | MON(10^9^/L) | 0.62 ± 0.24 | 0.65 ± 0.29 | 0.363 |
|  | EOS(10^9^/L) | 0.05 (0.02-0.11) | 0.06 (0.02-0.13) | 0.631 |
|  | BAS(10^9^/L) | 0.03 ± 0.02 | 0.03 ± 0.02 | 0.222 |
|  | PLT(10^9^/L) | 221.28 ± 74.90 | 216.07 ± 58.17 | 0.524 |
|  | SII | 1046.96 ± 860.57 | 1111.67 ± 799.92 | 0.505 |
|  | SIRI | 2.73 ± 1.97 | 3.30 ± 2.68 | 0.033 |
|  | MLR; | 0.35 ± 0.18 | 0.37 ± 0.22 | 0.282 |
|  | NLR | 4.75 ± 3.57 | 5.27 ± 3.73 | 0.227 |
|  | PLR | 129.77 ± 72.01 | 126.97 ± 62.70 | 0.720 |
|  | AISI | 600.27 ± 462.27 | 714.63 ± 630.50 | 0.068 |
| Post-PCI | WBC(10^9^/L) | 9.64 ± 2.34 | 10.63 ± 2.71 | 0.001 |
|  | NEU% | 75.48 ± 6.84 | 77.90 ± 7.61 | 0.005 |
|  | LYM% | 16.90 ± 5.43 | 14.89 ± 6.15 | 0.004 |
|  | MON% | 6.54 ± 2.02 | 6.41 ± 2.22 | 0.595 |
|  | EOS% | 0.50 (0.20-1.00) | 0.30 (0.10-0.80) | 0.018 |
|  | BAS% | 0.20 (0.10-0.30) | 0.20 (0.10-0.30) | 0.837 |
|  | NEU(10^9^/L) | 7.33 ± 2.11 | 8.35 ± 2.53 | ＜0.001 |
|  | LYM(10^9^/L) | 1.60 ± 0.58 | 1.53 ± 0.64 | 0.338 |
|  | MON(10^9^/L) | 0.62 ± 0.23 | 0.67 ± 0.28 | 0.074 |
|  | EOS(10^9^/L) | 0.04 (0.02-0.09) | 0.04 (0.01-0.08) | 0.070 |
|  | BAS(10^9^/L) | 0.02 (0.01-0.03) | 0.02 (0.01-0.03) | 0.474 |
|  | PLT(10^9^/L) | 212.06 ± 68.02 | 204.68 ± 55.24 | 0.327 |
|  | SII | 1093.81 ± 697.03 | 1337.09 ± 851.37 | 0.007 |
|  | SIRI | 3.17 ± 2.07 | 4.25 ± 3.02 | ＜0.001 |
|  | MLR | 0.42 ± 0.2 | 0.49 ± 0.25 | 0.015 |
|  | NLR | 5.21 ± 2.7 | 6.62 ± 3.96 | ＜0.001 |
|  | PLR | 147.08 ± 69.81 | 155.51 ± 73.06 | 0.321 |
|  | AISI | 599.06 (384.65-801.26) | 668.66 (432.92-1024.86) | 0.016 |
| Δ | WBC(10^9^/L) | -0.67 ± 2.25 | -0.46 ± 2.48 | 0.453 |
|  | NEU% | 4.38 ± 12.32 | 5.77 ± 15.76 | 0.391 |
|  | LYM% | -4.51 ± 10.17 | -5.67 ± 13.23 | 0.389 |
|  | MON% | 0.31 ± 2.40 | 0.49 ± 2.32 | 0.512 |
|  | EOS% | -0.08 ± 0.80 | -0.47 ± 1.34 | 0.005 |
|  | BAS% | -0.08 ± 0.27 | -0.11 ± 0.19 | 0.214 |
|  | NEU(10^9^/L) | -0.15 ± 2.52 | 0.21 ± 3.08 | 0.263 |
|  | LYM(10^9^/L) | -0.50± 1.02 | -0.63 ± 1.31 | 0.307 |
|  | MON(10^9^/L) | 0.00 ± 0.28 | 0.03 ± 0.25 | 0.406 |
|  | EOS(10^9^/L) | -0.01 ± 0.08 | -0.05 ± 0.13 | 0.006 |
|  | BAS(10^9^/L) | -0.01 ± 0.02 | -0.01 ± 0.02 | 0.172 |
|  | PLT(10^9^/L) | -9.22 ± 30.93 | -11.38 ± 25.38 | 0.527 |
|  | SII | 46.85 ± 847.46 | 225.42 ± 1057.89 | 0.104 |
|  | SIRI | 0.43 ± 2.41 | 0.95 ± 2.94 | 0.096 |
|  | MLR | 0.08 ± 0.21 | 0.12 ± 0.20 | 0.098 |
|  | NLR | 0.46 ± 3.85 | 1.35 ± 4.96 | 0.081 |
|  | PLR | 17.30 ± 71.32 | 28.54 ± 84.13 | 0.233 |
|  | AISI | 63.20 ± 505.14 | 164.59 ± 695.53 | 0.140 |
